# Human Respiratory Syncytial Virus Epidemiological Burden in Pediatric Outpatients in Italy: A Systematic Review

**DOI:** 10.3390/vaccines11091484

**Published:** 2023-09-14

**Authors:** Sara Boccalini, Benedetta Bonito, Cristina Salvati, Marco Del Riccio, Enrica Stancanelli, Mario Bruschi, Giulia Ionita, Johanna Iamarino, Davide Bentivegna, Primo Buscemi, Giulia Ciardi, Claudia Cosma, Lorenzo Stacchini, Cristiana Conticello, Manjola Bega, Annamaria Schirripa, Sonia Paoli, Lorenzo Bertizzolo, Salvatore Parisi, Francesca Trippi, Paolo Bonanni, Angela Bechini

**Affiliations:** 1 Department of Health Sciences, University of Florence, 50134 Florence, Italy; 2 Medical Specialization School of Hygiene and Preventive Medicine, University of Florence, 50134 Florence, Italy; 3 Sanofi, 14 Espa. Henry Vallée, 69007 Lyon, France; 4 Sanofi, Medical Affairs, Viale L. Bodio, 37/b, 20158 Milan, Italy

**Keywords:** RSV, ambulatory, epidemiology, children, hRSV-A, hRSV-B, respiratory infection, monoclonal antibodies, prevention, burden of disease

## Abstract

Background: Human respiratory syncytial virus (hRSV) is a key contributor to lower respiratory tract infections (LRTIs), affecting children aged 0–5 years and often leading to outpatient visits, emergency department utilization, and hospitalization. With the development of hRSV vaccines for mitigation, understanding the epidemiological impact of hRSV infections among 0–5-year-old pediatric outpatients in Italy is crucial. Methods: This systematic review conducted searches on PubMed, Embase, Scopus, and the International HTA Database, yielding 20,845 English and Italian records from January 2000 to July 2022. Results: Six eligible articles were identified following inclusion and exclusion criteria. These studies demonstrated hRSV-positivity proportions ranging from 18% to 41% in pediatric outpatients with respiratory infections. However, data comparability was hindered by diverse diagnostic approaches, data sources, sample populations, and study designs. Notably, hRSV-positivity showed temporal variability, rising from 23.8% (2001–2002) to 40.6% (2019–2020). This trend could stem from evolving epidemiological factors, heightened clinician awareness in hRSV diagnosis, or more sensitive molecular techniques. Conclusion: As the first review of its kind, this study underscores the need for more comprehensive data to inform effective preventive strategies against hRSV-related burdens in pediatric outpatients.

## 1. Introduction

Human respiratory syncytial virus (hRSV) is a recognized public health concern and a leading cause of hospitalizations due to lower respiratory tract infections (LRTIs) such as bronchiolitis and pneumonia in children < 5 years of age [1,2]. While hRSV disease is usually mild and self-limiting and presents the typical symptoms of upper respiratory tract infections (URTIs) like cough and cold [3], it can progress to more severe LRTIs requiring medical attention. Although nearly all infants contract hRSV by the age of 2 years, more than 20% of them may develop respiratory diseases necessitating medical assistance (emergency department and pediatric practice visits) [3,4], and 2–4% need hospitalization [5,6,7,8]. hRSV is the primary cause of LRTIs in children, responsible for up to 80% of bronchiolitis-related and 40% of pneumonia-related hospitalizations [9,10]. Severe hRSV disease may be characterized by symptoms such as tachypnoea and dyspnea, frank hypoxia, and cyanosis; physical examination might reveal wheezing, rales, and ronchi. Children born prematurely or with severe conditions like immunosuppression or congenital heart disease are at higher risk of severe hRSV disease [3]. Furthermore, it is important to note that getting infected with the virus (or developing a hRSV-related disease) does not always ensure complete protection against future infections [11]. Young age (below 6 months) at the start of the hRSV season is a major risk factor for severe hRSV-associated infections. Additional risk factors include preterm birth and respiratory or cardiac comorbidities (i.e., congenital heart disease), while the most susceptible groups to develop a severe disease are immunocompromised subjects or patients with cystic fibrosis or those with chromosomal abnormalities such as Down syndrome [8,12,13,14,15,16,17,18].

The only antiviral agent licensed for the treatment of severe hRSV infections is aerosolized Ribavarin; however, its use has been limited to immunocompromised patients [19] due to the cost of the treatment, potential toxicity, teratogenic effects, and the need for hospital admission for prolonged administration [20]. Due to the lack of an effective therapy, the reduction in morbidity and mortality from hRSV mostly relies on preventive measures [13]. Currently available preventive tools include two monoclonal antibodies (mAbs): Palivizumab (Synagis^®^) [21] and Nirsevimab (Beyfortus^®^), the latter of which was recently authorized by the FDA and EMA (European Medicines Agency, Amsterdam, The Netherlands) [22]. In Italy, Palivizumab is reimbursed for all preterm children < 29 weeks of gestational age (wGA), those <6 months of age at the beginning of each hRSV season, and those <2 years of age born with certain additional risk factors, such as cardiac heart disease and chronic lung disease [23]. Nirsevimab (Beyfortus^®^) is the first long-acting mAb designed to protect against hRSV-LRTI in all newborns and infants entering their first hRSV season, and this mAb is available in a single dose that induces protection that lasts for the entire hRSV season (at least 5 months) [24]. As already recommended by Italian Scientific Societies, Nirsevimab should be included within the national immunization calendar to implement preventive strategies that can reduce the burden of hRSV-LRTIs, including pediatric outpatients [25,26]. Furthermore, other preventive approaches are under investigation in different clinical trial phases, such as pediatric and maternal vaccines and other mAbs [27]. In addition to these recommendations, recent developments have expanded preventive options for specific populations. Two hRSV vaccines, RSVPreF3 (Arexvy^®^) and RSVpreF (Abrysvo^®^), have been approved for use in adults aged 60 years and older in Europe [28,29]. These vaccines offer protection against severe LRTIs caused by hRSV in individuals 60 years and older. Moreover, Abrysvo^®^ has gained EMA approval for use in pregnant women between 32 and 36 weeks of gestational age to prevent LRTIs and severe LRTIs caused by hRSV in infants from birth through 6 months of age. This significant development marks the first vaccine approved for use in pregnancy to protect infants from hRSV-related disease.

Given the availability of the above-mentioned preventive tools, including potential new additions, it is essential to gather data to better understand their possible applications. Therefore, the primary objective of this systematic review is to offer an epidemiological overview of hRSV infection in pediatric outpatients aged 0–5 years old in Italy. These data will contribute to the existing knowledge base, enabling more informed decision-making in the future.

## 2. Methods

### 2.1. Protocol and Registration

A systematic review was performed in accordance with Preferred Reporting Items for Systematic Reviews and Meta-Analyses (PRISMA) guidelines (Supplementary File S1) [30]. The review protocol was registered in the PROSPERO international database for systematic reviews on 8 August 2021 (registration number CRD42021248309).

### 2.2. Record Study Search

Reviewers searched for publications from 1 January 2000 to 14 July 2022 in PubMed, Embase, Scopus, and the International HTA Database. The PICO Framework was applied to identify the words of the query search.

The following search strategy was used and adapted to the consulted databases: (RSV OR hRSV OR “Respiratory Syncytial Virus” OR bronchiolitis OR ILI OR ARI OR SARI OR respiratory infection OR “respiratory tract infection” OR RTI OR URI OR URTI OR LRI OR LRTI OR “Viral pneumonia” OR otitis) AND (burden OR impact OR epidemiol* OR economic OR cost* OR hospital* OR incidence OR prevalence OR diagnos* OR diagnosis OR “laboratory confirm*” OR surveillance) AND (Italy OR Italian OR Italians OR Ital*) AND (paediatric OR child* OR toddler* OR newborn* OR infant* OR preterm OR pediatric*). All original studies published in the English and Italian languages were searched, and the record extraction was performed on 14 July 2022.

### 2.3. Inclusion and Exclusion Criteria

Studies focusing on hRSV infection in an outpatient pediatric population (0–5 years old children, up to 60 months) in Italy during the time 2000–2022 were included. Both observational studies and clinical trials were included. Reviews, letters, posters and conference abstracts, as well as all records that did not meet the inclusion criteria, were excluded from the final database. We also excluded studies referring to only hospitalized patients.

### 2.4. Screening and Study Selection

All extracted studies were collected in an Excel (Microsoft Excel^®^ per Microsoft 365 MSO © Microsoft 2022 Microsoft Corporation, Washington, DC, USA) file, and duplicates were removed. Titles, abstracts, and full texts were screened before eligibility assessment by four reviewers, working in couples, in a double-blind way. Possible disagreements were resolved by a fifth investigator. The reference lists of all eligible papers and previously published literature reviews were inspected to find other additional articles covering the same topic.

### 2.5. Data Retrieval and Analysis

After completing the record selection process, all articles were read and fully analyzed. Reviewers extracted the following data from each record: (1) General information of the study: authors, title, year of publication; (2) Study design (cohort, case-control, cross-sectional studies); (3) Geographical context and time of observation; (4) Characteristics of participants: age group, sample size; (5) Clinical and epidemiological outcomes: clinical diagnosis, prevalence of hRSV infections, percentages of laboratory-confirmed hRSV infection (genotyping of hRSV-A and hRSV-B, when available), differential diagnosis by specifying which other respiratory viruses involved, possible coinfections and hRSV seasonality. Among the retrieved articles, only those reporting specific data on pediatric outpatients were analyzed for this systematic review. The results of our systematic review are presented narratively and accompanied by tables and figures. 

### 2.6. Quality Assessment of the Included Studies

The quality assessment of the included studies was conducted by adapting a tool designed by Li and colleagues [1] (Supplementary File S2). Based on the assessment of different questions (on study testing, subjects, case definition, sampling strategy, etc.), an overall score was calculated.

## 3. Results

### 3.1. Selection Process

**Figure 1:**
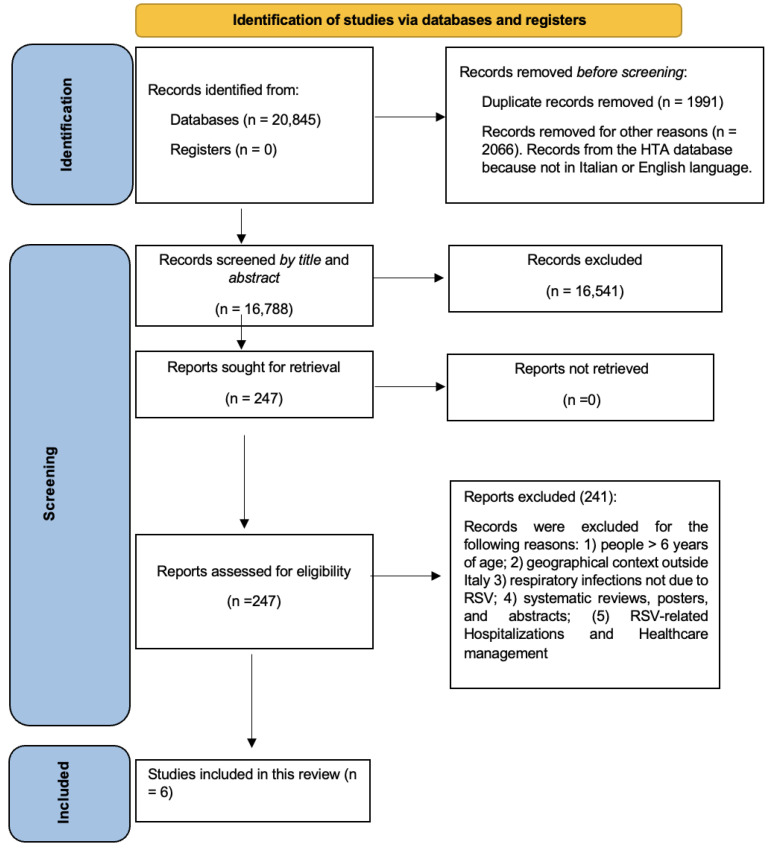
Flow diagram for the systematic review.

A total number of 20,845 records were downloaded from the cited databases. Figure 1 shows the flow-chart that describes the article selection process. Duplicates (n = 1991) and other records (2066), neither in the Italian nor the English language, were removed; 16,788 records were screened by title and abstract, and 16,541 were excluded, with 247 papers finally read in full. Subsequently, 241 articles were further excluded according to the above-mentioned criteria: i.e., children > 6 years old, geographical context outside of Italy, respiratory infections not due to hRSV, period other than 2000–2022; hRSV-related hospitalizations and healthcare utilization. This yielded six articles, which were analyzed considering the pediatric outpatient epidemiological burden.

### 3.2. HRSV-Associated Epidemiological Burden in Italy

Overall, six studies on hRSV-associated burden in outpatients in Italy were included in this systematic review (Table 1). These studies collected data from children affected by influenza-like illness (ILI), acute respiratory infection (ARI), acute respiratory tract infection (ARTI), severe acute respiratory tract infection (SARI) and LRTI, referring to a period from 2001 to 2021 [31,32,33,34,35,36]. All six studies included in this systematic review were found to be of high quality during assessment (Supplementary File S3). 

**Figure 2:**
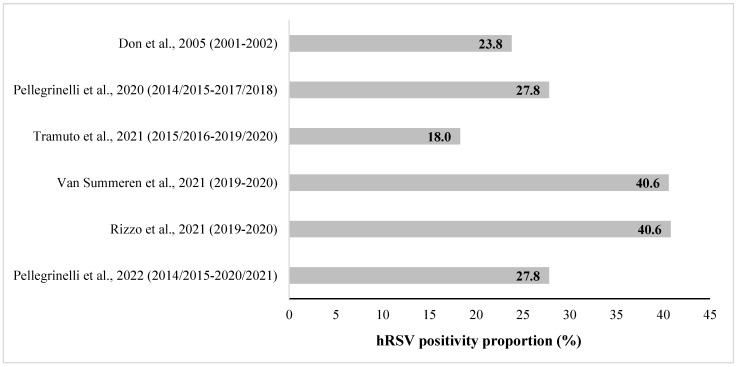
hRSV-positivity proportion (%) in pediatric outpatients aged ≤5 years old in Italy, reported in the retrieved studies. [Note: partial overlapping population for the studies of Pellegrinelli et al., 2020 and Pellegrinelli et al., 2022; overlapping population for the studies of Van Summeren et al., 2021 and Rizzo et al., 2021].

The proportion of hRSV-positive subjects varied among the outpatients: particularly, Don et al. [31] reported an hRSV-positivity proportion of 23.8% in outpatients < 5 years old; Tramuto et al. [33] found an hRSV-positivity proportion ranging from 17.6% to 19.1% in children < 5 years of age; and Pellegrinelli et al. in 2020 [32] and 2022 [36] reported an hRSV-positivity proportion 27.8% in the age group 0–5 years. Van Summeren et al. [34] and Rizzo et al. [35] showed an hRSV-positivity proportion of 40.6% in children <5 years old (Figure 2).

Don et al. [31] performed a serological survey in pediatric subjects with community-acquired pneumonia in Udine (Northern Italy) in both hospitalized patients and outpatients in the period 2001–2002. In particular, 66.7% (42/63) of children aged <5 years old were treated as outpatients. The authors highlighted the cases in which the etiology of the infectious disease was of bacterial/viral or mixed nature. Moreover, 23.8% of all community-acquired pneumonia in outpatient children up to 5 years old was due to hRSV. The other agents found in coinfection were *Streptococcus pneumoniae* (9/42; 21.4%), *Mycoplasma pneumoniae* (3/42; 7.1%), and *Chlamydophila pneumoniae* (1/42; 2.4%). Lastly, 30.9% of cases reported mixed infections or unknown etiology. 

Data by Pellegrinelli et al. (2020) [32] were retrieved from the database of virological influenza surveillance of the regional reference laboratory for Lombardy (Northern Italy) among the general population, operating within the Italian influenza surveillance network (InfluNet). The authors reported an overall hRSV-positivity proportion of 27.8% (51/183) in ILI cases of children aged ≤ 5 years old in four winter seasons (from 2014–2015 to 2017–2018). Particularly, in the different seasons, they observed an hRSV-positivity proportion of 34.8% in the 2014–2015 season, 15.2% in the 2015–2016 season, 34.2% in 2016–2017, and lastly, 32.5% in 2017–2018. Moreover, they molecularly characterized the hRSV infection and they found out that hRSV-B prevalence was significantly higher than hRSV-A in the four consecutive winter seasons: 16.7% of hRSV-A vs. 83.3% of hRSV-B (*p* = 0.04) [32].

In 2022, Pellegrinelli et al. (2022) [36] updated the previous study [32] by extending the observation time to seven seasons, up to 2020–2021. Overall, the hRSV-positivity was confirmed to be 27.8% in children ≤ 5 years of age. Particularly, the hRSV-positivity proportion among the different seasons observed was: 35.6% in 2014–2015, 16.9% in 2015–2016, 31.6% in 2016–2017, 33.3% in 2017–2018, 39.6% in 2018–2019, 31.3% in 2019–2020 and 0% in 2020–2021. Interestingly, the authors also stratified the hRSV cases among different age groups. No hRSV case was found in the 0–3-month-old population, and a decreasing hRSV trend was found along with increasing age: 36.4% in the 4–6-month-old population; 29.2% in the 7–12-months-old population; 27.6% in the 13–24-month-old population; and finally, 27.9% in children 25–60 months old [36].

Tramuto et al. [33] reported more data on hRSV epidemiology on ILI outpatients and SARI hospitalized cases in Sicily (Southern Italy) in the general population, which were analyzed together. hRSV more frequently spread among children < 5 years of age with an overall hRSV-positivity ratio of 18% (470/2609), ranging from 17.6% in children 2–4 years old to 19% in those aged < 12 months and 19.1% in those aged 12–23 months. Moreover, the genotype distribution of hRSV-B and hRSV-A was as follows: 50.9% (239/470) of hRSV-A, 46.6% (219/470) of hRSV-B, and 2.5% (12/470) of both hRSV-A + hRSV-B. In addition, results were also stratified by age (including both outpatients and hospitalized patients): patients ≤ 11 months (232 subjects) were slightly more infected by hRSV-A (56.8%) than hRSV-B (43.2%); while in the 1–4 years old subjects, no significant difference was reported between the hRSV-A and hRSV-B distribution. Moreover, in this last age group, they found coinfections of hRSV-A + hRSV-B: 1.9% in 12–23-month-old subjects and 3.1% in the 2–4-year-old children.

The multicenter and prospective study of Rizzo et al. [35], conducted between November 2019 and March 2020, provides an overview of the burden of hRSV-related ARI diagnosis in young outpatients (aged < 5 years) enrolled in Lazio (Central Italy) and in Puglia (Southern Italy). hRSV cases found were 119/293 (40.6%), and the age distribution of hRSV-positivity proportion reported was as follows: 40.8% in infants aged 0–12 months, 37.1% in toddlers aged 13–24 months, and finally, 43% in children 25–60 months. This study also shows the ARI case distribution caused by other infectious agents: hRSV was the second-most-detected agent, while Rhinovirus accounted for 43.7% of cases, Adenovirus for 10.2% and Bocavirus for 8.2% of cases, respectively. Moreover, Van Summeren et al. [34], analyzed the same Italian population as Rizzo et al. [35], reporting additional information on the hRSV genotype. Among the 119 hRSV-positive cases, hRSV-A accounted for 76% (91/119) and hRSV-B for 24% (28/119). In detail, hRSV-A was detected in 75% (40/53) of infants aged 1–12 months and in 81% (21/26) of toddlers aged 13–24 months. Finally, the hRSV-A-positive proportion in children 2–4 years old was 75% (30/40). Among the total hRSV-positive subjects, 1% (1/119) had chronic respiratory disease, 2% (2/119) had other chronic medical conditions, and 5% (6/119) were premature [34,35].

## 4. Discussion

This systematic review analyzed six studies that focused on the epidemiological burden of hRSV among pediatric outpatients aged 0–5 years (up to 60 months) in Italy from 2001 to 2021. The hRSV-positivity proportion among the studies retrieved showed a wide range in this age group, spanning from 18% to 41%, and data were not easily comparable. These variations were influenced by numerous factors, primarily linked to study design, recruitment methods, observation periods, age groupings, and geographic locations. As a matter of fact, in the selected studies, we found that different inclusion criteria for the diagnosis of CAP, ILI, ARI, and SARI were adopted to recruit the outpatient population. It is noteworthy that the selected studies utilized diverse study design: Don et al. [31], Van Summeren [34] and Rizzo [35] conducted prospective studies, while Pellegrinelli et al. (2020 [32] and 2022 [36]) and Tramuto [33] performed retrospective analyses. Moreover, these last three studies reported data retrieved from the Italian Surveillance Network for Influenza (InfluNet), which is not specifically designed to collect hRSV data, while the other studies acquired data from specifically designed projects.

Lastly, other characteristics not related to the study design have also been shown to be associated with results assessment: in some cases, the authors reported data from children up to 4 years, while Pellegrinelli 2020 [32] and 2022 [36] also included data from 5-year-old children. Moreover, Tramuto [33] and Rizzo [35] stratified the results by age sub-groups not fully comparable with each other. Finally, the sample size could also affect the results of each study. As a matter of fact, Don et al. [31] enrolled 42 outpatient children aged < 5 years in one season, while Tramuto et al. [33] enrolled a total of 2609 children aged 0–4 years in five seasons.

It is important to note that the hRSV-positivity proportion in pediatric outpatients seems to have increased over time. In the past (2001–2002 season), it was found to be 23.8% of outpatients < 5 years old (Don et al. [31]). More recently, Rizzo et al. [35] and Van Summeren et al. [34] showed an hRSV-positivity in children <5 years old of 40.6% (although the sample population of these two studies may overlap) in the 2019–2020 season. On the other hand, Pellegrinelli et al. 2020 [32] and 2022 [36] reported an average hRSV-positivity proportion of 27.8% in the 0–5 years age group, with a trend of higher annual values over time. This observed trend in hRSV-positivity in outpatients may have different explanations. First, general practitioners and pediatricians have become more aware of hRSV-related respiratory disease, and this could have led to higher attention in identifying the cause of respiratory infection. Further, improved diagnostic tests have been developed: during the early 2000s, diagnosis was performed by chest radiography and enzyme immunoassays [31], whereas nowadays, the more sensitive molecular characterization by RT-PCR is routinely used in many laboratories, which is also able to distinguish between the hRSV-A and hRSV-B subtypes. To gain a more comprehensive understanding of the virus’s spread and implications, it is imperative to enhance our monitoring and surveillance efforts. Moreover, taking into account the potential influence of climate factors such as temperature and humidity, as documented in recent studies, may further enrich our comprehension of hRSV seasonality [37,38].

However, things changed in 2020 throughout the first stage of the COVID-19 pandemic, when interventions such as social distancing and the usage of face masks reduced viral transmission, leading to a reduction in hRSV cases worldwide [39]. As expected, as COVID-19 restrictions eased between March and July 2021, hRSV resurfaced first in the southern hemisphere [40], where it was winter, before making a similar resurgence in the northern hemisphere, including Europe [41]. Currently, the circulation of hRSV has increased, especially during the last two northern hemisphere winter seasons (2021–2022 and 2022–2023) [42].

The studies retrieved in this systematic review do not cover the season 2022–2023; however, for that season, the Istituto Superiore di Sanità (Italian National Institute of Health, Rome, Italy) in Italy monitored (and continues to monitor) the hRSV circulation for the first time through the national ILI surveillance network InfluNet [43]. The cumulative incidence of ILI cases requiring medical assistance was very high in the 0-4 years age group, peaking in late November–December (cumulative incidence of 714/1.000). Many of the ILI syndromes in children have been caused by hRSV: 49.1% in the <2 years age group and 22.3% in the 2–4 years group [43]. Moreover, the two hRSV subtypes, A and B, have circulated differently over the years. However, hRSV-B seems the one more linked to severe respiratory infections that may have led to hospitalization [33]. Van Summeren et al. [34] compared hRSV data between Italy and The Netherlands, and they found comparable results in both countries: hRSV-A accounted for 76% and 75% in Italy and the Netherlands, respectively. However, among the general population, the prevalence of hRSV by subtype varied during the seasons observed: hRSV-B significantly predominated over hRSV-A during both the 2014–2015 and 2017–2018 seasons, accounting for 81.2% and 87.1% of all cases, respectively (*p* < 0.001). However, hRSV-A was most frequently detected in the 2016–2017 season, accounting for 62.5% of all cases vs. 37.5%; (*p* = 0.02). Finally, in the 2015–2016 season, hRSV-A and hRSV-B co-circulated at similar frequencies: 46.2% vs. 53.8% (*p* = 0.5) [34].

Another relevant issue to highlight is related to the health conditions of children aged ≤ 5 years with hRSV respiratory infection. Most cases of hRSV infections happen in otherwise healthy children [44], while children with pre-existing pathologies (such as cardiovascular diseases, chronic respiratory diseases, metabolic diseases, and immunodeficiencies) had about a 19-fold increased risk of contracting an hRSV infection than an influenza virus infection [36].

Our review faces some limitations. Firstly, the small number of included studies restricts the generalizability of our findings. Additionally, the extended timeframe covered by these studies poses challenges in assessing potential temporal variations in hRSV infection patterns in Italy. These variations may be influenced by external factors like temperature and humidity and by human factors, including possible changes in clinical practices and healthcare service organizations. Nonetheless, the geographic specificity provides a unique opportunity to offer in-depth insights into hRSV infection within Italy’s pediatric population, even though the findings may have limited generalizability beyond this context.

## 5. Conclusions

To our knowledge, this is the first review to collect hRSV data about the pediatric population of outpatients in Italy, and this may be considered an additional reason to implement further studies. The results of our systematic review found that hRSV in Italy greatly contributed to respiratory infections in pediatric outpatients aged 0–5 years old. Our results could probably underestimate the real epidemiological burden in Italian pediatric outpatients due to the self-limiting nature of the hRSV disease and its underdiagnosis. In the future, more data on this topic will be crucial, including the implementation of an ad hoc national surveillance system for hRSV infection or the strengthening of the existing national surveillance of ILI (InfluNet). Country-specific data availability on the epidemiology of hRSV will be essential for designing the most appropriate and effective immune-prophylaxis strategies to reduce hRSV disease burden in pediatric outpatients. In the future, prospective preventive strategies for all neonates and infants entering their first hRSV season, who are the most vulnerable and more likely to require medical assistance due to an hRSV-LRTI (such as bronchiolitis and pneumonia), should be implemented. The inclusion of monoclonal antibodies within the national immunization calendar, as already recommended by Italian Scientific Societies [45], may facilitate the implementation and maximize the equity of hRSV prevention for all neonates and infants entering their first hRSV season.

## Figures and Tables

**Table 1:** Characteristics of the studies retrieved, including hRSV-positivity proportion in outpatients.

Author	Region/City	Time of Observation	Case Definition	Age (as Reported by Each Study)	hRSV-Cases/Total (n/N)	hRSV-Positivity Proportion (%)
Don M, 2005 [31]	Udine (city, Northern Italy)	Season 2001/2002	CAP	<5 years	10/42	23.8
Pellegrinelli L, 2020 [32]	Lombardy (region, Northern Italy)	From 2014/2015 to 2017/2018	ILI	≤5 years	51/183	27.8
Tramuto F, 2021 [33]	Sicily (region, Southern Italy)	From 2015/2016 to 2019/2020	ILI, SARI	0–11 months 12–23 months 2–4 years	44/232 107/561 319/1816	19.0 19.1 17.6
Van Summeren JJGT, 2021 [34]	Lazio (region, Central Italy) and Puglia (region, Southern Italy)	Season 2019/2020	ARI	<5 years	119/293	40.6
Rizzo C, 2021 [35]	Lazio (region, Central Italy) and Puglia (region, Southern Italy)	Season 2019/2020	ARI	0–12 months 13–24 months 25–60 months	53/130 26/70 40/93	40.8 37.1 43.0
Pellegrinelli L, 2022 [36]	Lombardy (region, Northern Italy)	From 2014/2015 to 2020/2021	ILI	≤5 years	103/370	27.8

Abbreviations: ILI: influenza-like illness; ARI: acute respiratory infection; SARI: severe acute respiratory infection; CAP: community-acquired pneumonia.

## Data Availability

Data supporting reported results are available upon request to the corresponding author. Data were collected and managed in aggregated form according to European Union Regulation 2016/679 of European Parliament and the Italian Legislative Decree 2018/101.

## Supplementary Materials

The following supporting information can be downloaded at: https://www.mdpi.com/article/10.3390/vaccines11091484/s1, Table S1: PRISMA Checklist; Table S2: Quality scoring criteria; Table S3: Quality assessment score of individual studies included in the analysis
