# Supplementary material for: Human Respiratory Syncytial Virus Epidemiological Burden in Pediatric Outpatients in Italy: A Systematic Review
Source: Vaccines (Basel). 2023 Sep 14;11(9):1484. doi: 10.3390/vaccines11091484 (PMC10534716; doi:10.3390/vaccines11091484)
Supplement: Supplementary file 1 [file vaccines-11-01484-s001.zip › Supplementary File S2.pdf]

Supplementary File S2 Table S2: Quality scoring criteria

**Category**	**Questions**	**Scoring criteria**
Study design	Q1. Whether cases were prospectively enrolled?	• Yes – **1 point**<br>• No/unclear – 0 points
Study subjects	Q2. Any subgroup(s) exclusion that may have affected estimates?	• No – **1 point**<br>• Yes/unclear – 0 points (e.g., excluding neonates)
Case definition	Q3. Whether common/standard definitions were used?	• Yes – **1 point**<br>• No/unclear – 0 points
Sampling strategy	Q4. What is the proportion of eligible cases that were tested for RSV?	• ≥90% – **1 point**<br>• <90% but a systematic sample of eligible cases were tested – **1 point**<br>• <90%/unclear – 0 points
Diagnostic test	Q5. Whether PCR was used for the confirmation of RSV infection?	• Yes – **1 point**<br>• No/unclear – 0 points
Statistical analysis	Q6. Was there appropriate statistical analysis, including all needed adjustments?	• Yes – **1 point**<br>• No/unclear – 0 points
