# Supplementary material for: Human Respiratory Syncytial Virus Epidemiological Burden in Pediatric Outpatients in Italy: A Systematic Review
Source: Vaccines (Basel). 2023 Sep 14;11(9):1484. doi: 10.3390/vaccines11091484 (PMC10534716; doi:10.3390/vaccines11091484)
Supplement: Supplementary file 1 [file vaccines-11-01484-s001.zip › Supplementary File S3.pdf]

Supplementary File S3 Table S3: Quality assessment score of individual studies included in the analysis

References	Study period	Quality assessment score
Don M, 2005[29]	Season 2001/2002	0.8/1.0
Pellegrinelli L, 2020[30]	From 2014/2015 to 2017/2018	0.8/1.0
Tramuto F, 2021[31]	From 2015/2016 to 2019/2020	0.8/1.0
Van Summeren JJGT, 2021[32]	Season 2019/2020	1.0/1.0
Rizzo C, 2021[33]	Season 2019/2020	1.0/1.0
Pellegrinelli L, 2022[34]	From 2014/2015 to 2020/2021	0.8/1.0
